# A Tale About the First Weights and Measures Intercomparison in the United States in 1832

**DOI:** 10.6028/jres.111.003

**Published:** 2006-02-01

**Authors:** Albert C. Parr

**Affiliations:** National Institute of Standards and Technology, Gaithersburg, MD 20899-8440

**Keywords:** Hassler, history, intercomparison

## Abstract

In preparation for the Hassler memorial tablet dedication in the National Institute of Standards and Technology (NIST) Administration Building lobby in December of 2004, I learned that Ferdinand Rudolph Hassler had carried out the first systematic study of weights and measures in the United States. I obtained a copy of *Comparison of Weights and Measures of Length and Capacity Reported to the Senate of the United States* which is Hassler’s 1832 report to Congress on this comparison that related all the weights and measures then in use by the states and federal customhouses. Handwritten inscriptions in the book reveal interesting facts about Hassler’s work and his communication with other scientists in Europe at that time.

## 1. Introduction

Ferdinand Rudolph Hassler immigrated to the United States in the late summer of 1805 with his family and other Swiss emigrants, seeking to start a new life and escape the conflicts brought on by the French revolution in the late 18th century. Hassler, born in 1770 in Aarau, Switzerland, was a trained scientist with expertise in surveying and astronomy, and an accomplished mathematician. His family was prominent in Aarau and their financial position and local influence enabled Hassler to utilize his considerable talents in a variety of government service positions. Among them was the task of surveying local districts for the Swiss government. He had extensive travels in Europe prior to his immigration to the United States and, due to his travels and accomplishments, was known to a number of prominent European scientists and scholars. When he immigrated to the United States he brought a large scientific library and a number of high quality surveying instruments as well as standards of mass, length and volume that were accurate replicas of those being used in Europe in the late 18th and early 19th centuries [1,2].

Hassler died in 1843 and as a tribute to his leadership and accomplishments the members of the Coast Survey erected a stone monument in the late 1840s at the Laurel Hill Cemetery in Philadelphia where Hassler was buried. The Hassler family and friends replaced the original stone with a new stone in 1993 due to the fact that the original stone was showing signs of weathering after 150 years of exposure to the elements. The original commemorative stone was donated to NIST for its Museum collection. In addition to his duties as head of the Coast Survey, Hassler started the Bureau of Weights and Measures at the direction of President Jackson in 1830. When the Bureau of Standards was started by congress in 1901, it assumed the functions of the Bureau of Weights and Measures that was a part of the Coast Survey. In his role as the first Superintendent of the Bureau of Weights and Measures, Hassler must be considered the originator of the standards and measurement work that NIST now performs. It is a fitting tribute to Hassler’s contribution to weights and measures in the United States that NIST was able to place the historic commemorative tablet in the Administration Building Lobby in a December 2004 ceremony. As chair of the NIST Museum Committee, I participated in the preparation of this ceremony and became better acquainted with Hassler’s contributions to science in the early United States.

Hassler’s many accomplishments and the numerous problems he overcame are well documented in his biographies [1,2]. Additionally, the Treasury Department’s Coast Survey preserved many of his official letters and reports that were published as Weights and Measures documents [3]. The NIST Research Library has a copy of some of the Weights and Measures Documents for the early Coast Survey, which contains Hassler’s reports and correspondence with his colleagues. In particular, the collection of documents for the period of 1832 to 1845 covering the time of the weights and measures comparison greatly aided the efforts reported here.

**Fig. 1:**
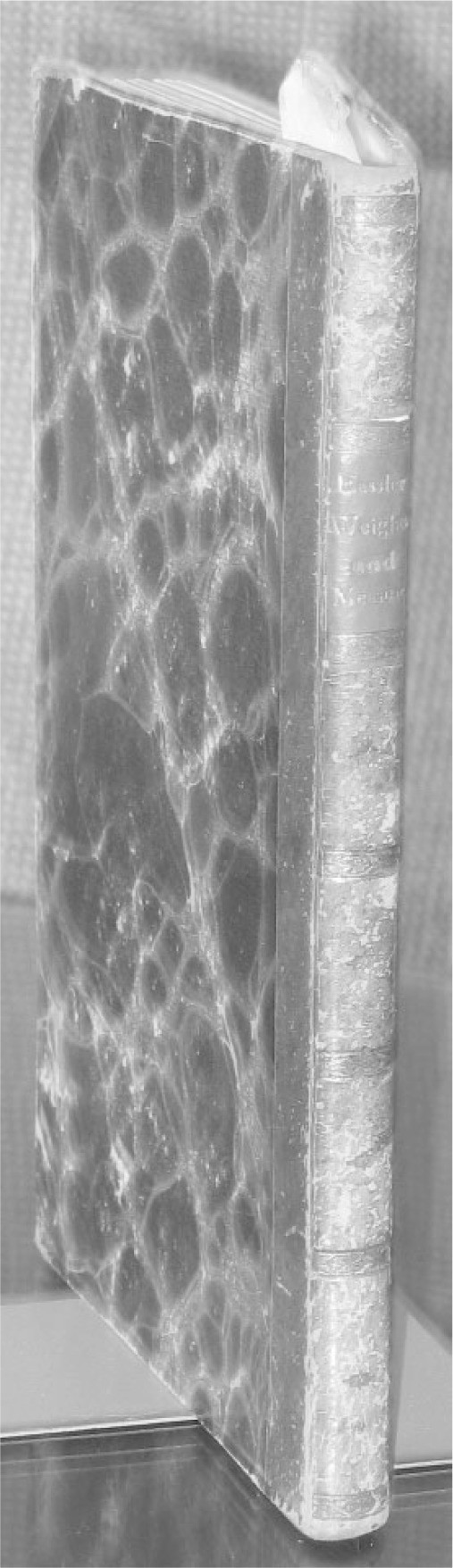
Photograph of Hassler’s report on weights and measures titled, Comparison of Weights and Measures of Length and Capacity, Reported the Senate of the United States by the Treasury Department in 1832.

Hassler carried out the first survey and comparison of weights and measures in use in the early United States and provided a report to Congress in 1832 that was presented through the Secretary of the Treasury under whose department Hassler was assigned [4]. These reports were printed as a part of the Congressional Record and copies were made available to Hassler. Hassler communicated directly with the Secretary of the Treasury, the President, and members of Congress as the occasion required. During his service to the country in the early 19th century Hassler was able to meet directly with the various presidents and treasury secretaries as needed. Much of this official correspondence is preserved in the Coast Survey documents. While participating in the installation of the Hassler tablet in the Administration Building lobby and preparing the dedication ceremony, I became interested in getting a copy for my personal library of Hassler’s 1832 report on the weights and measures comparison. I located and purchased a copy on the web that was advertised by a bookseller in Germany. The book, shown in Fig. 1, turned out to have some interesting historical tales that offer insight into the activities of Hassler and others engaged in early measurement and standards work. This paper highlights the interesting historical connections discovered about this particular book.

**Fig. 2:**
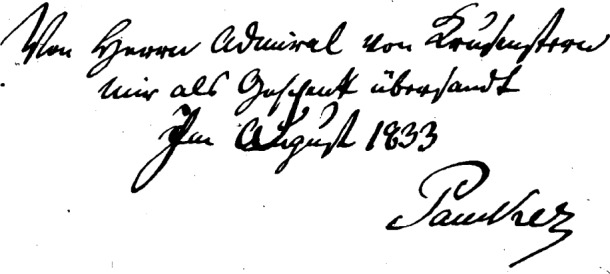
Inscription inside the front cover of the book shown in Fig. 1.

The book has two hand-written inscriptions, one on the inside front cover and the other on the title page. Figure 2 is the inscription on the inside front cover and reads in German:

Von Herrn Admiral von Krusenstern mir als Geschenk übersandt Im August 1833 Pauker

Translated into English this inscription says:

“Sent to me as a present by Admiral von Krusenstern in August 1833 Pauker”

**Fig. 3:**
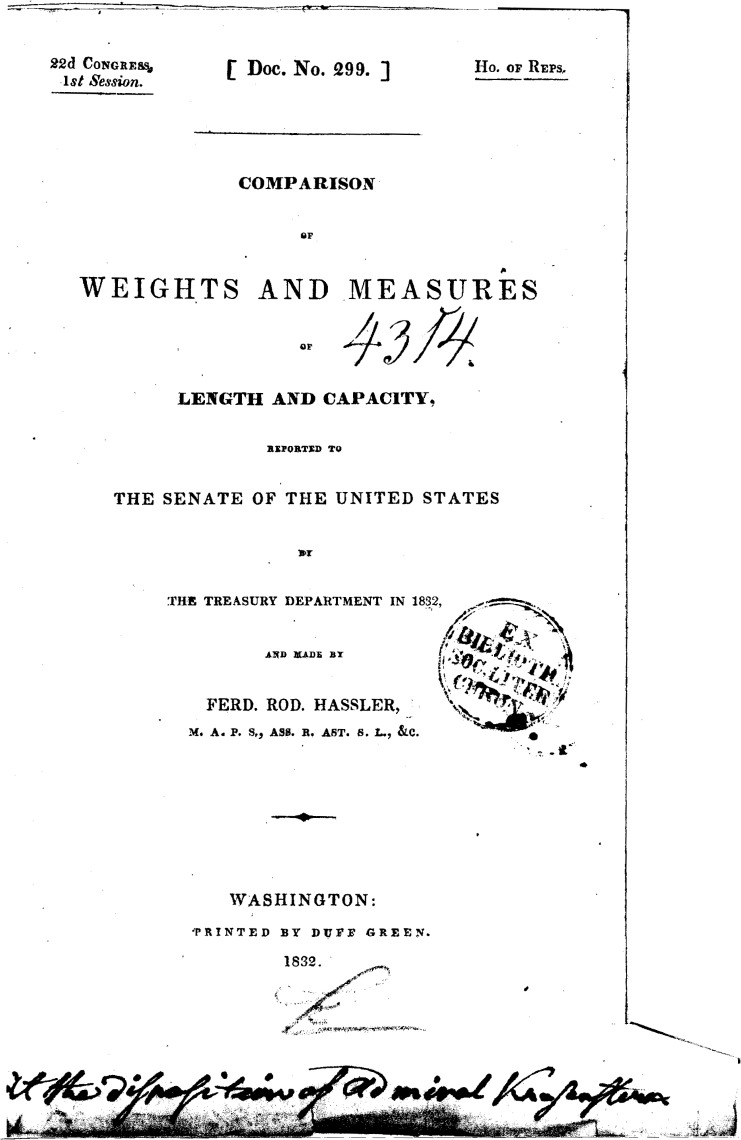
Title page of book shown in Fig. 1 that has inscription by the author and a library stamp.

Figure 3 shows the inscription on the title page of the report. It is in English and says:

“At the disposition of Admiral Krusenstern”. (Note: In early printed and written English the letter “s” was often written as “f” as shown in Fig. 3)

With the help of colleagues at NIST, I have managed to piece together what these inscriptions imply about Hassler’s communication with other scientists of the time, and place this particular copy of the Weights and Measures report within its historical context. Dr. Alfons Weber, a Scientist Emeritus in the Optical Technology Division, has been a critical and essential participant in this story as he deciphered the old German script in the book as well as other early German material we obtained elsewhere. I am indebted to his help in unraveling this fascinating story about early metrology in the United States. Ms. Harriet Hassler of the NIST library has been extremely helpful in locating material about her distant relative that has been invaluable for this effort.

## 2. The Principal Persons Involved in the Story

The people whose names appear in the two inscriptions, Krusenstern and Pauker, were contemporaries of Hassler and were well-known scientists who both had an interest in mathematics, surveying, and weights and measures. Brief histories of them and of Edward Troughton, who is mentioned in correspondence we obtained between Hassler and Krusenstern, are given.

## 3. Krusenstern

**Fig. 4:**
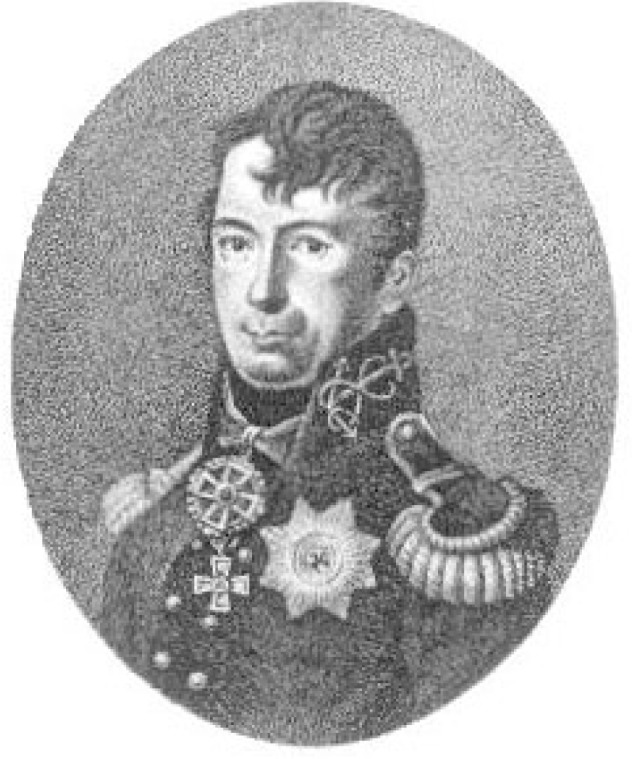
Likeness of Admiral Krusenstern of the Russian Navy obtained from the following website: http://www.mala.bc.ca/~black/amrc/krusen.htm. This website is maintained by Professor John Black, Malaspina University College, Nanaimo, B.C., Canada, and this image is used with his permission.

Adam Ivan Krusenstern was a well known Russian navigator and admiral born in Estonia in 1770, the same year as Hassler, but spent most of his professional life in the service of the Russian Navy [5]. Krusenstern (also spelled Kruzenshtern) had interests in geography and surveying, as did Hassler. Krusenstern is most noted for his 1803–1806 circumnavigation of the earth, the first for a ship of the Russian Navy. During this voyage he made numerous stops for scientific and cultural exploration, including Pacific islands and Japan, and published a narrative of his journey and observations which became a reference for diverse scientific fields from anthropology to surveying of coastlines [6]. A likeness of Krusenstern in Naval uniform is shown in Fig. 4. Krusenstern and Hassler corresponded as a result of meeting each other when they were both in London in 1814-15. Hassler’s biographer Cajori mentions the meeting, which is also mentioned by Krusenstern in one of the letters, preserved in the Coast Survey documents. In a letter dated March 6, 1831 in response to a letter from Hassler, Krusenstern says:

“I remember very well that I made your acquaintance in 1814 and ’15 at old Mr. Troughton’s, and frequently I have tried to obtain information on your intended Survey of the Coast of America, for which you had the instruments made in London, …”

With the help of Dr. Alexander Prokhorov, a guest scientist in the Optical Technology Division who comes from Russia, we obtained copies of letters written by Hassler to Krusenstern during the period of 1831 to 1841 from the Russian Naval Archives in St. Petersburg. In addition to the letters from F. R. Hassler, the archives furnished a copy of a letter from Hassler’s son to Krusenstern informing the Admiral of his father’s death in November of 1843.

Hassler had gone to London at the request of the United States Government in August of 1811 to procure instruments for surveying the coast of America. Hassler had a long and troubled relationship with the United State Government over the execution of the Coast Survey, which vexed him throughout most of his life in the United States. Cajori’s biography details some of the troubles Hassler had over the Coast Survey as does the material in the Coast Survey documentation [1,3]. However these tribulations are not the focus of the present discussion, which is focused on Hassler’s interaction with Krusenstern. Hassler and Krusenstern were both in London at the same time procuring a wide range of surveying instruments including transits, sextants and heliotropes for use in their respective countries. This trip took Hassler much longer than anticipated and was made more difficult by the outbreak of the War of 1812. Hassler returned to the United States with his instruments and other scientific items in 1815 upon the cessation of hostilities between Britain and the United States.

## 4. Troughton

Edward Troughton was born in 1753 and joined his older brother John in a scientific instrument maker’s shop in London that the older Troughton had started. John died in 1788 and Edward assumed the management of the shop and was the principal designer and engineer. The brothers had built a precision dividing engine that enabled them to provide the highest quality sextants and other precision instruments for surveying and navigation [7]. Edward continued to build the business after his brother’s death and established a reputation as the best instrument maker in England if not all of Europe. He constructed instruments for the leading scientific organizations at the time and his fame and expertise is what brought both Hassler and Krusenstern to London in 1814 to procure precision instruments for their work. Troughton also manufactured standard weights and standards of length. Hassler procured some of these items and they became the property of the Bureau of Weights and Measures in the Coast Survey and some now reside in the NIST Museum. Troughton retired from the firm in 1826 due to failing health and died in 1835. The firm continued for many years as Troughton and Simms and later as Cooke, Troughton and Simms, until the operations of the firm were merged into larger businesses in the mid 20th century.

## 5. Pauker

Magnus Georg Pauker (also Paucker) was a contemporary of Krusenstern and Hassler being born in 1787 and dying in 1855 [8]. He graduated from Dorpat University, now called Tartu University, in Estonia and had similar interests to Krusenstern and Hassler. He participated in land surveys and taught mathematics at the Academia Petrina. Pauker also participated in weights and measures intercomparisons as we learn below from Krusenstern’s letter to Hassler. In this letter Krusenstern informed Hassler on how he had distributed the reports on weights and measures that Hassler had sent him. Pauker also was instrumental in organizing the first scientific society in Latvia, called the Kurland Society, and published various mathematically related papers in Latvia and Russia. Pauker, together with Krusenstern and a number of other prominent scientists whose families had immigrated to the Baltic States from Germany were collectively were known as the Baltic Germans. There was a large German influence in the Baltic area beginning in the Middle Ages as a result of the spread of Christianity and to satisfy German interests at increasing commerce in the Baltic area. This influence grew when Peter the Great of Russia encouraged commerce with northern Europe, in part to help establish a Russian Navy based in St. Petersburg. Peter and some of the later Tsars encouraged German migration to Russia to facilitate the improvement of Russian technology and decrease Russia’s traditional isolation from the west. The Germans were granted a great deal of autonomy and established German language institutions in the Baltic and in Russia. This relationship lasted until the late 19th century when the political climate changed in Russia and a great many of the ethnic Germans left.

## 6. Weights and Measures Report

Prior to the inception of the income tax in the early 20th century, the United States Government was financed by duties on imports and exports. A reliable and uniform system of weights and measures was important for gathering taxes as well as settling commercial disputes. The federal government maintained a system of customhouses for determining appropriate import and export taxes on items of commerce. In his report to Congress, Hassler lists 47 customhouses for which he compared the bushel used for volume determinations. Hassler found the mean volume to be 2153 cubic inches, with variation as much as almost 100 cubic inches. He compared the various units of length in use at that time in United States commerce including the French toise, the Troughton standard scale of about 82 English inches, and various European length scales. Hassler had procured the Troughton standard for the Coast Survey in London during his stay of 1811-15. He constructed a dividing instrument to accurately divide up the length units in order carry out the measurements. Hassler also developed and built barometers and thermometers so that appropriate barometric and temperature corrections could be made to the measurement. He measured coefficients of expansion and other pertinent thermal properties of the materials he used in the construction of his instruments to assess temperature effects on the measurements. It is interesting to note that Hassler was able to borrow from the American Philosophical Society in Philadelphia some of the standards that he sold to them when he first arrived in the United States in 1805. He was forced, shortly after arriving in the United States, to sell many of his instruments and standards as well as a portion of his library material to support himself and his family. Hassler and his Swiss emigrant colleagues were the victims of some sort of misappropriation of their funds by a land agent whom they had relied upon to buy them land in Georgia for a new Swiss settlement. Hassler and the other Swiss never made it to Georgia as a group and Hassler spent his life in the pursuits we have mentioned.

Hassler had brought to America, when he emigrated in 1805, a newly constructed meter that the French had produced as a result of the survey of France in the 1790s [9]. The meter was defined as one ten-millionth of the distance from the equator to the pole and hence ambitious surveys were carried out by the French and others to obtain this quantity precisely. The first meter bar was called a Committee Meter since it was defined by a committee of French scientists. During the upheaval of the French revolution many aspects of French life were governed by committees who performed functions previously the responsibility of the King’s appointees. The Committee Meter that Hassler brought to the United States is on display in the NIST Museum.

To carry out his comparison of weights, Hassler constructed instruments to augment those he purchased abroad. Among the items constructed were some mercury balances which functioned by displacement of mercury by a large float attached to a weighing pan for the unknown weight. The device was calibrated with known weights prior to use. For less massive weights, water could be used as the liquid in this type of device. Hassler set up instrument shops in Washington DC and employed and trained instrument makers to construct instruments and weight and volume sets for the various states as required by his charge from Congress. He adopted the troy pound of Great Britain as the standard of weight for these measurements. The Mint had a copy of this standard manufactured at the request of the United States Government in 1824 [4]. Hassler realized the importance of knowing accurately the expansion properties of water and mercury and performed experiments on the substances to determine the appropriate coefficients. Hassler was able to make excellent measurements; for example he derives the yard from the Troughton scale as 36.0002465 inches. This number indicates the precision that Hassler tried to achieve in his comparison of the weights and measures.

The report to Congress contains all the results from the various customhouses for both their volumetric and length standards. Additionally Hassler generated tables that allowed the conversion of the various units used at the time among themselves. For example he gives the “authentic meter of the committee” as equal to 39.3842349 inches at a temperature of 39.8 degrees Fahrenheit. The comparison work provided data for the relationships of the weights and measures from the various customhouses for all of their measurements, including the ounces, grains, bushels, and other units commonly then in use.

For his effort as the superintendent of the Bureau of Weights and Measures, Hassler was paid $3000 per year [3]. He was given additional allocations to purchase instruments and hire assistants. $3000 per year was a significant salary in the mid 1800s. Hassler paid his assistants much less, less than $1000 per year in some cases. When he was heading the Coast Survey in 1840, he was paid $4500 per year and assistants were paid from $780 to as much as $4000. In some cases these people had to support themselves while on the field surveys and pay for other expenses associated with the survey. The cost of the survey and his spending were issues that caused friction between Hassler and Congress throughout his career and led to many disagreeable exchanges between Hassler and various congressional and governmental officials.

The other major conflict he had was in the disagreement over the surveying technique used in the Coast Survey. Accurate chronometers had been designed in the 18th century for use in navigation and there were a number of people in the Navy department who proposed the chronometric technique for the coast survey [10]. With an accurate chronometer and sextants, one can, as does a navigator aboard a ship, determine the longitude of any position on the earth. The latitude can be determined with a sextant and a measurement of the angular position of Polaris, the North Star. Hassler conversely believed that the triangulation method used in land survey was most accurate and was the only one that could produce reliable and accurate maps. This disagreement about methodology caused the Coast Survey to be put in the Navy Department at times and then, when Hassler prevailed, put back in the Treasury Department. The story of Hassler’s difficulties is well documented by his biographer Cajori and is on an excellent website maintained by NOAA [1,11].

## 7. Hassler and Krusenstern

Hassler knew from his meeting with Krusenstern in London in 1814 that the Admiral was interested in surveying techniques since they were both at Troughton’s for the same purpose. The unit of length is of course critical to surveying and the relationship of the different units used throughout the world would naturally lead Hassler to assume that Krusenstern would be interested in his report on the weights and measures in the United States. Hassler wrote to Krusenstern in a letter dated January 2, 1833 that we obtained from the Russian Naval Archives:

“In the meantime I had the comparison of Weights and Measures of which I join here the report, in a number of copies to different directions, which Baron von Sacken is so kind to take under his charge, with the present, the inscriptions show their destination. (there are 7)”

The phrase, “in the meantime” refers to what he has been doing since the Coast Survey was removed from his control and placed with the Navy. Later in the letter, Hassler asks Krusenstern to convey one copy of the work to his old university friend, Mr. Bartels in Dorpat. Dorpat is the former name of what is now called Tartu in Estonia. Bartels was most likely Johann Martin Christian Bartels who was a friend of Karl F. Gauss and other mathematicians of the early 19th century. Bartels taught at Dorpat University and was an older student with Gauss when they were students together in Braunschweig, Germany [12]. Bartels’ daughter married the famous mathematician Friedrich Struve. I do not have good information on who Baron von Sacken was and how he fit into the picture other than acting as a courier for Hassler and Krusenstern. At this period in history the mail services were a complicated set of government and private courier arrangements and it was often difficult to arrange for packages and letters to be sent between countries. Exchange of mails between countries based upon stamps purchased at the origin did not become formalized worldwide until the Universal Postal Union was established in 1874. Certainly it was difficult for Hassler to send material from America to Europe at that time. The courier would have to be paid in advance and often the recipient was asked to pay fees as well. In one instance that Hassler refers to in his letters, he shipped some books to Europe but the customs in England where they first arrived did not get sufficient payment or there was some other discrepancy and the whole shipment was apparently burned.

Other parts of the letter deal with an effort by Hassler to sell Krusenstern copies of his mathematical works, primarily his logarithmic and trigonometric tables for use in the Russian Navy. After some effort, apparently this commercial endeavor worked out to everyone’s satisfaction. Hassler’s mathematical books gained widespread circulation and the money generated by sales was a source of income for the Hassler family between his appointments with the United States Government and other jobs he held.

In a letter dated July 11, 1833, Krusenstern states:

“Your letter dated New York, 2d January, reached me very late; it is not above six weeks since I received it, through Baron von Saken(Sacken). Pressing business on service, and an absence from St. Petersburg, have prevented me to answer sooner your letter, and to return my most obliging thanks for the copy of your Report on Weights and Measures, which you have done me the favor to send. The other six copies I have delivered to those persons to whom they were destined. One of the two copies you left to my option I shall send to Professor Pauker, at Mittau(Mitau), who has published lately a voluminous work on the same subject that has won the prize of Demidof, adjudged by the Academy of Sciences. He will consider your book a valuable gift; for it is indeed the production of a master, which reputation you deservedly enjoy in Europe as well as America.”

**Fig. 5:**
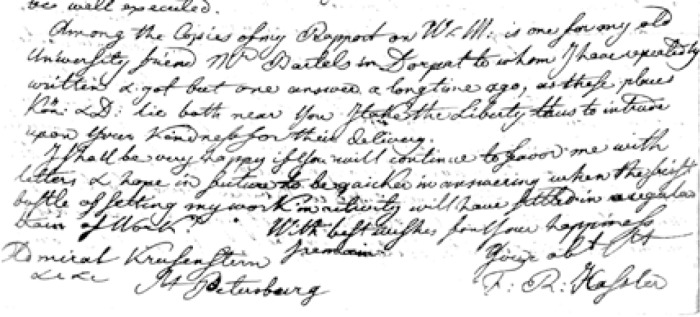
Copy of the last page of a letter Hassler sent Krusenstern on January 2, 1833 and which shows the writing of Krusenstern’s name is the same as the inscription in the figure.

This comment makes it clear that the copy of the Weights and Measures comparison that I obtained is one of the two that Hassler sent Krusenstern for his own use. The inscription shown in Fig. 3 is a dedication by Hassler that this volume is to be used for whatever purpose Krusenstern desires. Figure 5 is an excerpt from the letter that Hassler sent Krusenstern dated January 2, 1833. The excerpt is from the final page of the letter and shows Hassler’s signature as well as his address designating the recipient of the letter: Admiral Krusenstern of St. Petersburg. Comparing the style of the writing of Krusenstern’s name in the inscription in Fig. 2 and at the end of the January 2nd letter I obtained from the Russian Naval Archives makes it clear that Hassler, as he said in his letter to Krusenstern, inscribed on the volumes their intended destination. Krusenstern probably kept one of the two copies and sent the other, the one described here, to Professor Pauker. When I first obtained the book, I was mystified why the writing on the title page appeared to be of the same vintage as that on the inside cover but yet was in English when Pauker and Krusenstern would normally be expected to have written in German. It was a pleasant surprise to find the book is a dedication copy inscribed by the author, F. R. Hassler.

The title page that contains Hassler’s inscription as shown in Fig. 3 has a fold out portion that protrudes beyond the page boundary. This occurred, I would guess, because the book was probably printed on large paper stock with more generous margins and when it was bound, probably by Krusenstern or Pauker, the binder preserved the inscription by folding it in when he cut the margins to fit a smaller binding. Trimming a text block was a common practice of binders in the 19th century, and earlier as well, as it made the expense of the binding less and thereby saved the owner money. In early printing, books were often supplied by the printer to the author in a simple paper binding and the purchaser would have a cloth or leather binding made to suit his particular library. For the actual printing, the printer used whatever paper size was available to accommodate the page of text as laid out. In some cases this resulted in books with very wide margins that would be trimmed by the binder.

In a letter from Pauker to Krusenstern dated August 10, 1833, Pauker notes that he received the book of Hassler but was too busy to pursue it at the time. The letter is in German and is difficult to read because of the quality of the photocopy and the fact that in the original letter both sides were used. The ink of course penetrated the paper and makes an impression on both sides of the paper. This effect can be seen in the excerpt in Fig. 5. The bleed through of the ink makes photocopying difficult and since we do not have access to the originals, it is hard to decipher the full content of the letters. The Hassler letters to Krusenstern had similar problems, particularly the ones written in German. The Pauker letter discusses with Krusenstern his efforts in weights and measures and is about measurement comparisons in the Baltic area. Pauker’s other letters to Krusenstern are mainly about his efforts to have his son enrolled in the Russian Navy in order to become an officer. This, it seems, was eventually successful.

## 8. Conclusion

One of the pleasures resulting from book collecting is the unexpected information the collector often learns when pursuing a particular book and its origins. In the case of books that are dedication copies from the author to friends or colleagues, one can learn about the relationships and common interests that may have prevailed. In this case we learn that Hassler was acquainted with some of the most prominent surveyors and scientists of the time. His education and travel within Europe prior to his coming to the United States allowed him to become acquainted with many of the scientific leaders of Europe. In his biographical notes, Hassler says of his trip to Paris in 1794 [2]:

“I went to Paris, introduced myself to the Astronomer Lalande to Chevalier Borda, Astronomer Delandre and Lavoisier, collected a fine mathematical and diplomatic Library”

Jean-Baptiste-Joseph Delandre and his colleague Pierre-François-André Méchain were at that time involved in the survey of France in order to determine the meter. They were both trained by the Astronomer Joseph-Jêrôme Lalande[9]. These men were the leading astronomers in late 18th century France. Jean-Claude Borda was a naval officer and one of France’s most noted experimental physicists. Antoine Lavoisier was one of the most famous chemists of late 18th century France who among other things quantified the nature of chemical reactions. Lavoisier was from a wealthy family and was associated with some commercial endeavors, including that of belonging to the organization that collected taxes, that were in great disfavor by the revolutionaries in France and he suffered the fate of the guillotine in 1794. It was probably occurrences such as this which motivated many people, including perhaps Hassler, to seek a new life in America. Additionally the French Revolution spilled over into Switzerland and the uprisings there and Napoleon’s subsequent invasion in 1798 would have caused Hassler considerable consternation and cast a doubt about his continued technical work.

When Hassler came to the new world he had already made the acquaintance of some of the most influential scientists in Europe of his time. His trip to London and France during the War of 1812 allowed him to renew contacts with them and meet new people such as Krusenstern. These relationships gave Hassler a high level audience in Europe to communicate with and share information. His contacts enabled him to know which was the best instrument maker (Troughton) to make his surveying instruments and from whom to obtain accurate measurement standards. This relationship served the United States well as it enabled the early Bureau of Weights and Measures to obtain the best possible standards that were comparable to the best available to European nations. NIST scientists and engineers continue this legacy of providing the nation the best possible metrological tools.

The copy of the Weights and Measures Report that I obtained has had an interesting journey. It went from Washington DC where it was printed, to St. Petersburg, Russia in 1833. This journey took about 7 months and was carried for some part of the trip by the mentioned Baron von Sacken. How it was sent to Pauker is unknown but it was likely by someone carrying it to Mittau as a favor to Krusenstern. The Russian Admiral would not have found it difficult to find someone going by ship to Latvia. There is a library stamp on the title page that is from the library of the Kurland Society for Literature and Art which, as mentioned earlier, Pauker help start [13]. Pauker apparently contributed the book to the library of this society and it became a part of their holdings. It is the usual practice of libraries to occasionally pare their holdings down by selling infrequently used books or ones that no longer seem relevant. This book could have come from such a transaction but it will be difficult ever to tell.

In any event the book has returned home and hopefully can rest peacefully in the NIST historical collection in due course.
